# Constitutive volatiles as ecological synchronizers: is there a role for constitutive plant volatiles to optimize growth and defence in plant communities?

**DOI:** 10.1093/jxb/erag272

**Published:** 2026-09-01

**Authors:** Jurgen Engelberth

**Affiliations:** Department of Biology, Health, and the Environment, The University of Texas at San Antonio, TX, USA

**Keywords:** Adaptive plant growth, defense, gene expression, plant–plant communication, plant volatiles

## Abstract

This article comments on:

**Åbonde A, Rensing M, Gallinger J, Juárez-González VT, Dahlin I, Markovic D, Martinez G, Ninkovic V**. 2026. Volatiles released by undamaged plants mediate the adaptive growth strategies in neighbours. Journal of Experimental Botany **77**, 5124–5139. https://doi.org/10.1093/jxb/erag252

This article comments on:

**Åbonde A, Rensing M, Gallinger J, Juárez-González VT, Dahlin I, Markovic D, Martinez G, Ninkovic V**. 2026. Volatiles released by undamaged plants mediate the adaptive growth strategies in neighbours. Journal of Experimental Botany **77**, 5124–5139. https://doi.org/10.1093/jxb/erag252

**Åbonde *et al.* (2026) describe the effects of constitutive plant volatile organic compounds (cVOCs) from barley cultivars on the growth behaviour of other cultivars. cVOCs from fast-growing cultivars enhance growth of slow-growing cultivars, whereas cVOCs from slow-growing cultivars reduce growth of fast-growing cultivars. Furthermore, distinct cVOC bouquets were associated with cultivar-specific sets of genes. Although limited to different cultivars from one species, these findings imply that constitutive volatiles become integrated into the physiology of receiver plants and reallocate resources. While this has only been shown for cVOCs from conspecifics, cVOCs from other species may also contribute, thereby optimizing growth/defence allocations to provide the best possible survival strategies.**

Over the last three decades, volatile organic compounds (VOCs) produced by various organisms have been recognized as important regulators and modulators that help to facilitate interactions within and between different clades (Baldwin, 2010). As such, their ecological relevance is now well established, with new VOC-mediated interactions reported quite frequently. The relevance of VOCs was mostly established in the context of plant–herbivore interactions, where inducible VOCs (iVOCs) were found to be emitted by herbivory-damaged plants to attract natural enemies of the attacking herbivore. Furthermore, certain iVOCs also serve as signals between plants and provide significant protection in receiver plants by activating specific types of defences, often at a fitness cost. Major VOCs were identified as mainly belonging to the family of terpenes, with mono- and sesquiterpenes being the largest contributors, and so-called green leaf volatiles (GLVs) (Cofer *et al.*, 2018; Bouwemeester *et al.*, 2019; Hu *et al.*, 2021; Schuman, 2023), which were found not only to serve as anattractant for natural enemies, but also to provide protection to other plants nearby by priming their defences, so that they can respond faster and more strongly (Engelberth *et al.*, 2004). While these interactions were thoroughly characterized, certain bacterial VOCs can also influence plant physiology. Bacterial volatiles such as acetoin and 2,3-butanediol, both normally absent from plant-derived VOC bouquets, promote significant growth effects in plants (Ryu *et al.*, 2003; Fincheira and Quiroz, 2018). Bacterial VOCs can also induce systemic resistance, an effective defence response to pathogen attacks that also lacks major investments into direct defence but rather prepares the plant (Ryu *et al.*, 2004). Overall, these findings position VOCs as a versatile messenger that shapes ecological interactions across kingdoms.

However, little attention has been paid to VOCs that are constitutively emitted without any trigger by damage, herbivory, or pathogen infection. Usually, those volatiles are only measured to demonstrate inducibility of other volatiles, and no studies have—to the best of my knowledge—been performed beyond serving as controls. In the study by Åbonde *et al.* (2026), however, barley varieties with different growth characteristics were used to study the effects of constitutive VOCs (cVOCs) on conspecific growth. For example, the cVOC profiles from a slow-growing barley cultivar (Fairytale) were significantly different from those of a faster growing barley cultivar (Salome). Even more interesting is the finding that exposure of fast-growing Salome plants to cVOCs from slow-growing Fairytale plants reduced their growth, while exposure of Fairytale to cVOCs of Salome accelerated their growth. The differential growth was further supported by gene expression analysis, which also showed that different profiles were obtained by those treatments. Fairytale cVOCs up-regulated a very distinct transcriptional response in Salome, notably genes associated with RNA homeostasis and metabolism, while Salome cVOCs induced not only comparatively lower levels of transcript accumulation in Fairytale but also different categories linked to protein homeostasis and stress-related genes.

These contrasting responses of Salome and Fairytale cultivars to one another’s constitutive VOCs demonstrate that these VOCs have very distinct biological activities. Furthermore, most of these constitutive barley growth-promoting VOCs differ from those typically emitted by plants after herbivory or pathogen infections (Cofer *et al.*, 2018; Bouwemeester *et al.*, 2019; Hu *et al.*, 2021; Schuman, 2023). While some constitutive barley VOCs seem to be derived from fatty acids, allene oxide synthase (AOS) and hydroperoxide lyase (HPL) pathways do not seem to be involved. Other lipoxygenase (LOX) pathways also seem to be unlikely since those would result in a more distinct pattern of fatty acid-derived compounds depending on the specificity of the respective LOX. Also, linear alkanes such as tetradecane and dodecane, which are probably derived from the plant cuticle (Barcia *et al.*, 2007), are rarely found among pathogen- or herbivore-induced plant VOCs. These major differences between induced and constitutive VOCs may therefore be the key to explain their diverging activities in the setup used by Åbonde *et al.* (2026). However, a potential link that would allow for cVOCs from slow-growing cultivars to prepare receiver plants against incoming biotic threats was found. Some of the genes detected in these combinational treatments, including those encoding certain transcription factors, may be involved in some regulation of defences without actively increasing them, but potentially preparing receiver plants metabolically or through enhanced signalling for the activation of direct defences, which could also lead to a faster and stronger defence response if challenged, generally referred to as priming. Yet, since neither this novel way of priming nor the activities of individual compounds from these constitutive volatile profiles have been tested, current conclusions about the beneficial properties and their regulation at this time remain speculative. Yet, some protective effects by constitutive VOCs against aphids have been observed (Kheam *et al.*, 2023), but effects on other insect herbivores remain untested.

Among the constitutive volatiles identified, several were already known to affect growth behaviour in plants. Benzothiazole derivatives often have defensive properties and can affect growth (Simonova *et al.*, 2005; Dong *et al.*, 2021). Benzyl nitrile similarly affects plants by being an active defence compound but also stimulating growth (Urbancsok *et al.*, 2018; Liao *et al.*, 2020; Qian *et al*., 2023). Likewise, linalool and 1-octen-3-ol, primarily emitted by Salome, have been shown to activate defences and thus, also reduce growth in receiving plants (Pennerman *et al.*, 2022; Guo *et al.*, 2025). However, they are also found among the constitutive VOCs that increase growth in Fairytale. Therefore, just analysing the patterns of constitutive VOCs from these barley cultivars does not allow for any conclusions to be drawn as to which of these compounds might be the active ones. Targeted bioassays with individual compounds at proper ecologically relevant concentrations are therefore necessary to pinpoint the respective active compound(s) in the respective response. Comparative tests across species are required to determine whether this phenomenon is more general or limited to certain plant species.

The major question, however, is how the growth effects of these otherwise unremarkable compounds become integrated into the general physiology of receiver plants. Do they represent another tier of regulation reflecting environmental and ecological context, or do these VOCs directly interact with major growth-regulated hormone pathways as mediators that provide the necessary information regarding the environmental conditions? However, while some examples of this have been described (Dani and Loreto, 2022; Kanjana *et al.*, 2026), none of those studies used any of the cVOCs shown by Åbonde *et al.* (2026). Therefore, based on the current findings, it is more plausible that these cVOCs act through regular metabolic or VOC-specific signalling pathways rather than actively activating either growth or defences. This, however, can still lead to the observed effects, mainly through the differential modulation by these cVOCs of yet to be identified metabolic pathways.

By observing plants broadly across taxa, it appears that cVOCs convey information about the growth conditions of neighbouring individuals, primarily by eavesdropping, and regulate growth towards a more even appearance, thereby reducing competition which is otherwise regulated by the phytochrome system through shade avoidance, which in turn appears to also regulate cVOC emissions (Kegge *et al.*, 2015). Most species tested to date for their growth response VOCs belong to the family of *Poaceae*, which includes many turf, meadow, and prairie grasses, but also important crops such as maize, barley, wheat, and sorghum, which usually grow in dense stands. Equalizing growth in such populations may provide advantages for nutrient provision, herbivore defence, as well as other ecological factors. Even growth prevents individual dominance that would otherwise reduce resource availability for neighbours, and GLVs and other VOCs are likely to be much more effective when they accumulate within such a dense population and could thus provide much better protection. Conversely, rapid vertical growth by individuals could reduce the effectiveness of volatile defences by creating an increase in canopy heterogeneity, thereby creating gaps that facilitate pest and pathogen spread.

Cushion plants may illustrate an extreme example of this phenomenon: individuals maintain similar height, creating a compact canopy that buffers abiotic stress, traps heat, and may as such also permit VOC accumulation in the core. This may further enhance the effectiveness of both direct and indirect volatile defence signals. However, the precise regulatory mechanism producing this growth type remains unclear, but VOC-meditated interplant signalling may also be an important factor in this highly coordinated growth.

To synopsize, the implications of the results described by Åbonde *et al.* (2026) are outlined in Fig. 1, where community effects and the interaction of cVOCs with iVOCs are also shown in a model that may serve as a roadmap for future research. However, regardless of future findings, Åbonde *et al.* (2026) report another exciting phenomenon from the plant world, in which cVOCs from neighbouring plants regulate physiological responses of other plants by shifting the growth/defence balance to optimize survival. These findings underscore that plants in communities do not act solely according to fixed genetic programmes but integrate spatially and temporally variable environmental stimuli including constitutive volatile signals into their physiology to provide the best possible conditions for their success.

**Fig. 1.**
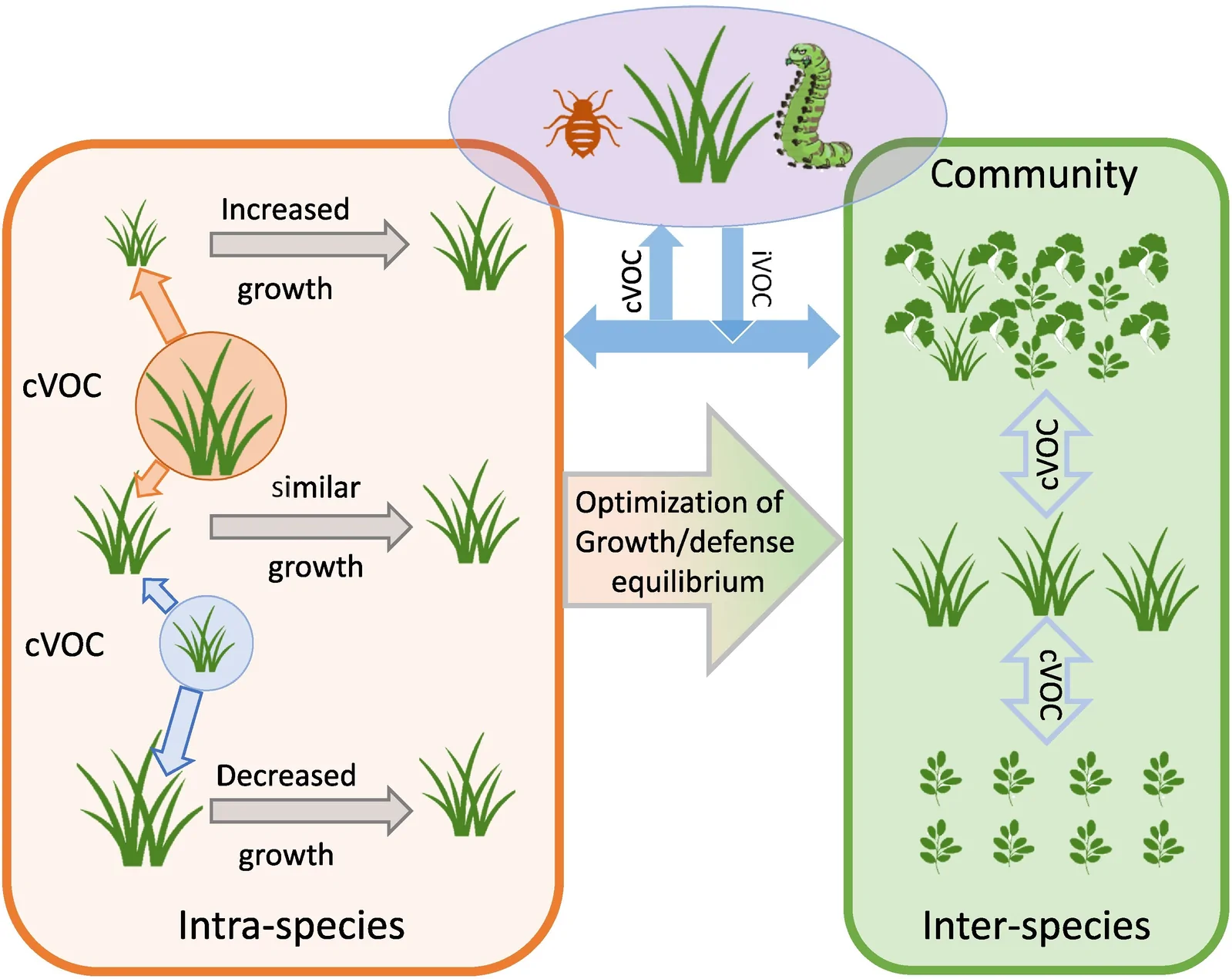
The integration of constitutive and inducible plant volatiles. Constitutive volatile organic compounds (cVOCS), as shown by Abonde *et al.* (2026) regulate growth–defense allocations in barley, resulting in adaptive growth behaviour (orange panel) and source-specific gene expression in receiver plants. Small cultivars exposed to specific cVOCS emitted by large cultivars (larger orange circle) increase growth, wheras large cultivars exposed to the cVOC bouquet of small cultivars (smaller blue circle) reduce growth. (Medium-growth cultivars did not show significant responses to either type of cVOC.) These specific growth responses should also have consequences for the cVOCs of the receiver plant. Since growth is adjusted to that of medium growth cultivars, will the cVOCs change accordingly? If not, what limits the effectiveness of cVOCs in receiver plants? I would hypothesize that such a change will occur if cVOCs are ecologically important cues for other plants and alter their responses. Answering those questions will provide important information about the regulation of cVOCs. Another important question is whether cVOCs from one plant species can also alter the behaviour of other plant species and consequently have relevant functions within plant communities. Plant–plant interactions have already been demonstrated across different species; however, those were mainly identical compounds. For cVOCs, we would hypothesize that those may also influence inter-species communication (green panel, right). If so, those interactions should affect whole plant communities. While these are important questions for research, further studies should also analyse the impact of cVOCs and induced VOCs (iVOCs) on each other (purple circle). Does cVOC communication affect the iVOC response? And can the cVOC bouquet be altered under the influence of iVOCs or is it even suppressed? Since these hypotheses are mainly anchored in the regulation of growth/defence allocations, this should have a significant impact on those responses, and consequently the interactions of plants under those respective conditions.
